# Induction of cross-reactive HIV-1 specific antibody responses by engineered V1V2 immunogens with reduced conformational plasticity

**DOI:** 10.1016/j.vaccine.2020.03.010

**Published:** 2020-04-16

**Authors:** Jennifer I. Lai, Susan K. Eszterhas, Seth A. Brooks, Chengzi Guo, Susan Zolla-Pazner, Michael S. Seaman, Chris Bailey-Kellogg, Karl E. Griswold, Margaret E. Ackerman

**Affiliations:** a Thayer School of Engineering, Dartmouth College, Hanover, NH, USA; b Department of Medicine, Division of Infectious Diseases, Icahn School of Medicine at Mount Sinai, New York, NY, USA; c Center for Virology and Vaccine Research, Beth Israel Deaconess Medical Center, Harvard Medical School, Boston, MA, USA; d Department of Computer Science, Dartmouth College, Hanover, NH, USA; e Department of Microbiology and Immunology, Geisel School of Medicine, Dartmouth College, Hanover, NH, USA

**Keywords:** Structure-based immunogen design, HIV-1 vaccines, V1V2 loops, Immunogenicity

## Abstract

Antibodies against the HIV-1 V1V2 loops were the only correlate of reduced infection risk in the RV144 vaccine trial, highlighting the V1V2 loops as promising targets for vaccine design. The V1V2 loops are structurally plastic, exhibiting either an α-helix-coil or β-strand conformation. V1V2-specific antibodies may thus recognize distinct conformations, and an antibody’s conformational specificity can be an important determinant of breadth and function. Restricting V1V2 conformational plasticity in an immunogen may thus provide control over the conformational specificity and quality of a vaccine-elicited antibody response.

Previously, we identified a V1V2 sequence variant (K155M) that results in enhanced recognition by cross-reactive antibodies recognizing the β-strand conformation. Here, we relate V1V2 antigenicity to immunogenicity by comparing the immunogenicity profiles of wildtype and K155M immunogens in two mouse models. In one model, immunization with gp70 V1V2 K155M but not wildtype elicited antibody responses that were cross-reactive to a panel of heterologous gp120 and gp140 antigens. In a second model, we compared the effect of K155M on immunogenicity in the context of gp70 V1V2, gD V1V2 and gp120, examining the effects of scaffold, epitope-focusing and immunization regimen. K155M variants, especially in the context of a gp120 immunogen, resulted in more robust, durable and cross-reactive antibody responses than wildtype immunogens. Restriction of the β-stranded V1V2 conformation in K155M immunogens may thus be associated with the induction of cross-reactive antibody responses thought to be required of a protective HIV-1 vaccine.

## Highlights

• K155M stabilizes V1V2 β-strand conformation relevant to broad antibody recognition.
• β-strand stabilized V1V2 immunogens elicit antibody responses with greater cross-reactivity.
• Fine-tuning immunogen conformation modulates immunogenicity.

## 1 Introduction

Despite decades of research, an effective vaccine for human deficiency virus type-1 (HIV-1) remains an elusive goal. Functional HIV-1 “cures” have thus far occurred only in very rare and specific situations [1], and there exist few generalizable examples of protective immunity against HIV-1. HIV-1 is also a master of immune evasion. A high mutation rate and ability to shield the envelope trimer glycoprotein via copious glycosylation and conformational masking due to structural dynamism [2], [3] result in a shape-shifting target for vaccine design. Thus, the path towards a vaccine for HIV-1 is as of yet unclear.

Recent isolation and structural characterization of broadly neutralizing antibodies (bnAbs) capable of potently neutralizing most HIV-1 strains has provided promising templates for vaccine design [4], [5], ushering in an age of structure-based vaccine design [6]. These efforts aim to utilize structural information of antibody (Ab):epitope recognition to recapitulate relevant epitopes in novel immunogens in order to selectively elicit a desired (bn)Ab response. However, though bnAbs are potent in *in vitro* neutralization assays and have shown promise as therapeutic and prophylactic biologic agents [7], [8], they have been challenging to elicit via vaccination [9]: bnAbs are usually isolated after years of infection, are not typically protective for the individuals from whom they were isolated, and often exhibit long complementarity determining regions and high levels of somatic hypermutation [4]. In the only modestly protective human vaccine trial to date (RV144) [10], [11], only Abs able to weakly neutralize Tier 1 viruses were detected following vaccination. Rather, analysis of correlates of protection pointed to a possible role for non-neutralizing Abs against the variable loops 1–2 (V1V2), perhaps via Fc-mediated recruitment of effector cells and Ab-dependent effector functions [12], [13], [14]. Together, these observations motivate a more holistic multifactorial model for vaccine-elicited Ab protection that may include both bnAbs and non-neutralizing yet broadly-reactive and functional Abs.

In addition to their potential role in the RV144 trial, V1V2-specific Abs are often detected during natural infection [15], [16], and there is evidence for immune pressure on V2 in both analyses of RV144 breakthrough viruses [17] and in longitudinal studies of virus-Ab co-evolution within individuals during natural infection [18], [19]. The immunological relevance of the V1V2 loops thus suggests a prominent role for anti-V1V2 responses and motivates V1V2 as a possible vaccine target.

Characterization of V1V2-specific monoclonal Abs (mAbs) has revealed three classes of V1V2-specific Abs, termed V2q, V2i and V2p, which differ in their modes of V1V2 recognition as well as in their neutralization potency, breadth and cross-reactivity. V2q mAbs, including quaternary-preferring bnAbs (e.g. PG9 and PG16), recognize the V1V2 loops with the V2 C-strand (V2C) in its constrained β-stranded conformation at the apex of the HIV-1 envelope trimer, and are among the most potent bnAbs reported [20], [21]. V2i mAbs (e.g. 697-30D and 830A) recognize a conformational epitope and also require the V2C β-strand conformation [22], [23], [24]. Though the V2i epitope appears to be mostly occluded in the closed pre-fusion envelope trimer [25], V2i mAbs have been shown to be widely cross-reactive against heterologous monomeric HIV-1 glycoprotein 120 (gp120) [15], and exert antiviral activity *in vivo* by decreasing viral load presumably through a combination of neutralizing and Fc-mediated effector functions [26]. Finally, V2p mAbs (e.g. CH58, CH59, CAP228-16H) recognize a linear peptide epitope and have been isolated from a recipient of the RV144 regimen [27], and from an HIV-1 infected donor [28]. Though V2p mAbs have been shown to mediate Ab-dependent cellular cytotoxicity [29], they are otherwise only weakly and narrowly neutralizing, and it has been suggested that the V2p epitope is only accessible on aberrant or misfolded envelope protein [23], [30]. Notably, V2p mAbs recognize diverse α-helix-coil V1V2 conformations (for example, mAbs CH58 and CH59 from the same individual recognize distinct V2 peptide conformations) [27], [28] that are likely incompatible with the β-barrel conformation required for V2q and V2i binding [20], [25]. Thus, despite the V1V2 loops’ propensity to adopt a conserved super-secondary structure, which may account for the cross-reactive nature of many V1V2 mAbs [24], they appear to dynamically ‘flicker’ between conformational states in which V2C assumes alternative β-strand or α-helix-coil states.

Given that V1V2 is structurally dynamic and that the V1V2 conformation that an Ab recognizes may modulate breadth and function [31], a structure-based V1V2 immunogen must exhibit conformational selectivity in order to elicit the desired conformation-dependent Ab response. In earlier work, we previously reported an HIV-1 V1V2 sequence variant K155M (HxB2 numbering, hereafter referred to as K-M) located in the conserved V1 β-strand of the V1V2 β-barrel [20], [25] that alters V1V2 antigenicity (Fig. S1) [32], with enhanced recognition by V2q/V2i mAbs and reduced binding by V2p mAbs across various scaffold and strain contexts, suggesting that K-M variants may favor the β-stranded conformation of the V2C strand.

Here, we first extend our characterization of K-M antigenicity utilizing a wider panel of mAbs as well as polyclonal Ab samples. Subsequently, we sought to determine whether the altered antigenicity of K-M variants would result in altered immunogenicity profiles with respect to Ab magnitude and breadth. We examined the effects of immunogen conformation (i.e. introduction of K-M), epitope-restriction and vaccine prime/boost regimen in two mouse models, and assessed plasma Ab binding to a panel of heterologous HIV-1 antigens as a readout for the development of cross-reactive Ab responses.

## 2 Results

### 2.1 V1V2 K-M variants exhibit altered antigenicity

#### 2.1.1 K-M results in differential binding to V1V2 and CD4 binding-site (CD4bs) mAbs

**Fig. 1:**
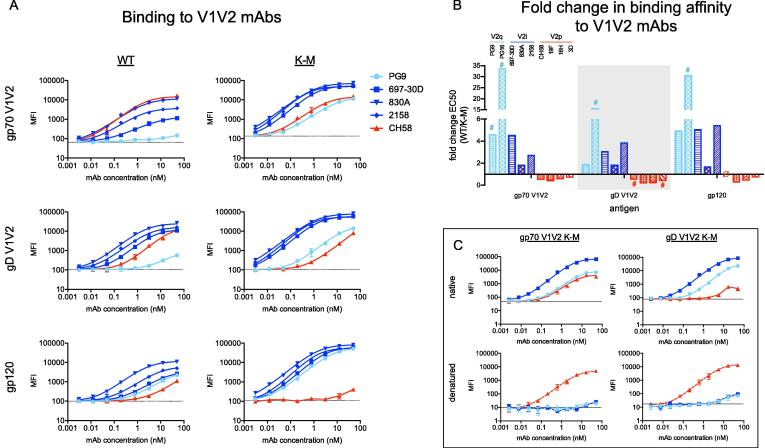
V1V2 K-M variants of gp70 V1V2, gD V1V2, and gp120 exhibit altered antigenicity to V1V2 mAbs with conformational and linear epitopes. A. Binding of WT and K-M V1V2 variants to a panel of V1V2 mAbs [V2q: PG9 (light blue); V2i: 697-30D, 830A and 2158 (dark blue); V2p: CH58 (red)]. B. Fold change in binding of K-M variants to an extended panel of V1V2 mAbs. Fold change was calculated as ratio of WT/K-M EC_50_ values as calculated by non-linear sigmoidal curve fits in GraphPad Prism. Values > 1 indicate enhanced K-M binding, while values < 1 indicate decreased K-M binding. For cases in which sigmoidal curve fits were ambiguous, EC_50_ values were bounded at the highest concentration tested (50 nM) to determine the WT/K-M ratio (denoted by “#”). Where neither WT nor K-M EC_50_ could be fit unambiguously (CH58 binding to gp120 antigens), “∅” is used. C. Binding of native (top, solid lines) and denatured (bottom, dashed lines) gp70- and gD- scaffolded V1V2 K-M to V1V2 mAbs. Ab classes are colored throughout as follows: V2q (light blue), V2i (dark blue), V2p (red). Dotted lines on y-axes represent background signal from no antibody controls, and error bars represent standard deviation of triplicate measurements. Nonlinear fit lines calculated in GraphPad Prism are shown for data where calculated EC_50_ values are non-ambiguous. (For interpretation of the references to colour in this figure legend, the reader is referred to the web version of this article.)

Using a more comprehensive panel of V1V2 mAbs than previously, we first assessed the antigenicity of strain BG505 V1V2 K-M variants in three scaffold contexts: gp70 V1V2 characterized previously, herpes simplex virus (HSV) glycoprotein D (gD)-scaffolded V1V2 (gD V1V2) in which V1V2 loops C-terminally are fused to the first 27 amino acids of HSV gD [33], and full-length monomeric HIV-1 glycoprotein 120 (gp120) (Table S1). Consistent with previous observations, all three K-M variants showed enhanced binding to V2q mAbs (PG9, PG16) [20] and V2i mAbs (697-30D, 830A, 2158) [34], and reduced binding to V2p mAbs (CH58, CAP228-16H, CAP228-19F, CAP228-3D) [27], [28], relative to the corresponding wildtype (WT) (Fig. 1A-B, Fig. S2, Table S2). Heat denaturation of gp70 V1V2 K-M and gD V1V2 K-M eliminated nearly all binding to PG9 and 697-30D, suggesting loss of the β-stranded V2C conformation necessary for binding by these mAbs (Fig. 1C). Notably, binding to CH58 increased after heat denaturation, with an increase in maximum signal intensity at saturation [1.2-fold (gp70 V1V2 K-M) and 31.7-fold (gD V1V2 K-M) after background subtraction] rather than a shift in apparent affinity [EC_50_ = 6.99 nM (native), EC_50_ = 7.05 nM (denatured) for gp70 V1V2 K-M], consistent with increased prevalence of the V2p state after denaturation rather than a change in the inherent binding affinity. Thus, V1V2 loop conformation in the non-denatured K-M variants may be constrained such that V1V2 conformation is skewed more towards β-strand than α-helix-coil, and disruption of the V1V2 β-barrel by denaturation may increase the availability of the V2p epitope.

**Fig. 2:**
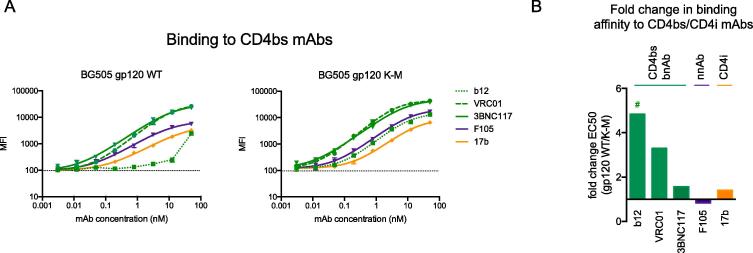
V1V2 K-M may alter binding of CD4bs mAbs in the context of gp120. A. Binding of gp120 WT and K-M to a panel of CD4bs and CD4i mAbs [CD4bs bnAbs in dark green: b12 (dotted), VRC01 (dashed), 3BNC117 (solid); CD4bs non-neutralizing F105 (purple); CD4i mAb 17b (orange)]. B. Fold change in binding affinity of gp120 K-M to CD4bs/CD4i mAbs relative to WT. Fold change was calculated as ratio of WT/K-M EC_50_ values as calculated by non-linear sigmoidal curve fits in GraphPad Prism. Values > 1 indicate enhanced K-M binding, while values < 1 indicate decreased K-M binding. The EC_50_ value for gp120 WT to mAb b12 was ambiguous and was bounded by the highest concentration tested (50 nM) to determine the WT/K-M ratio (denoted by “#”). Dotted lines on y-axes represent background signal from no antibody controls, and error bars represent standard deviation of triplicate measurements. Nonlinear fit lines calculated in GraphPad Prism are shown for data where calculated EC_50_ values are non-ambiguous. (For interpretation of the references to colour in this figure legend, the reader is referred to the web version of this article.)

Given that K155 is located on the underside of the V1V2 loops when mapped onto the BG505 SOSIP crystal structure [35] (Fig. S1, PDB ID: 5V8M), and that previously we only assessed binding of K-M variants to anti-V1V2 mAbs in the context of scaffolded V1V2 loops, we next sought to determine whether K-M affects the antigenicity of the CD4 binding site (CD4bs) in the context of monomeric gp120. We assessed binding of gp120 WT and K-M to a panel of CD4bs mAbs (reviewed in [36]) including bnAbs (b12, VRC01, 3BNC117), non-neutralizing mAb F105, and mAb 17b, which recognizes a CD4-induced (CD4i) epitope (Fig. 2A, Table S3). Binding to bnAb b12 was greatly enhanced by the introduction of K-M in comparison to gp120 WT, for which binding was minimal. Apparent binding affinity for VRC01 was additionally enhanced upon introduction of K-M relative to gp120 WT. Differences in binding affinity to other mAbs tested were less pronounced (Fig. 2B). Notably, CD4bs mAbs bind gp120 with varying angles of approach, and among the CD4bs bnAbs tested, b12 and VRC01 bind gp120 angling towards V1V2 at the trimer apex, while 3BNC117 approaches the CD4bs from below, angled away from the trimer apex (Fig. S3). CD4bs mAbs thus may vary in their potential to clash with the V1V2 loops, perhaps providing a structural hypothesis for the differential effect of K-M on CD4bs bnAb recognition. Thus, manipulations of the V1V2 loops such as K-M reported here or others [37], may also alter the accessibility of distal epitopes like the CD4bs.

#### 2.1.2 V1V2 K-M exhibits conformation-dependent antigenic characteristics with respect to polyclonal anti-HIV-1 Ab samples

**Fig. 3:**
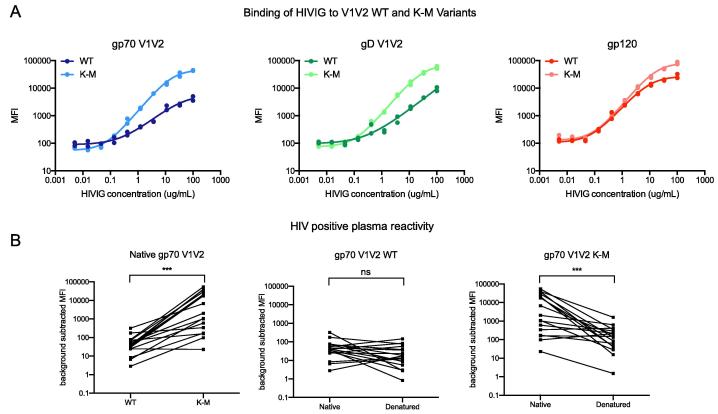
V1V2 K-M variants exhibit differential antigenicity to polyclonal anti-HIV antibody samples. A. Binding of WT and K-M variants to HIV immunoglobulin (HIVIG). Each of two replicates at each concentration step is shown, with a non-linear curve fit (Sigmoidal, 4 parameter logistic regression performed in GraphPad Prism). B. Binding of human HIV-positive plasma samples at a 1:2500 dilution (n = 17) to gp70 V1V2 WT and K-M (left), and after heat denaturation (center and right). Points represent the mean of three replicate measurements with background signal subtracted, and Wilcoxon matched-pairs signed rank test was performed in GraphPad Prism (***: *P* < 0.001).

In addition to altered V1V2 antigenicity with respect to mAb binding, we sought to determine whether K-M also imparts altered antigenicity with respect to polyclonal Ab samples representative of humoral responses to HIV-1 infection. To do so, we first probed the reactivity of pooled immunoglobulins from asymptomatic HIV-infected individuals (HIVIG) (Fig. 3A) to WT and K-M V1V2 variants. HIVIG binding to gp70 V1V2 K-M and gD V1V2 K-M variants resulted in roughly a 10-fold increase in the binding signal relative to the corresponding WT probe at the highest HIVIG concentration. Though maximum signal intensity at saturation can differ across antigen-conjugated beads based on the quantity of antigen coupled, all beads were conjugated in reactions with equivalent bead:antigen mass ratios. Thus, the quantity of antigen conjugated is not likely to fully account for the differential binding of HIVIG to WT and K-M probes on the same gp70 or gD scaffold; rather, the increased magnitude of binding to K-M probes may indicate that WT and K-M probes bind differentially to anti-V1V2 Abs present in HIVIG, with a greater quantity of Abs reactive to the K-M variants. In the context of gp120, HIVIG showed a less pronounced increase in binding to K-M compared to WT, likely because Abs recognizing non-V1V2 epitopes on gp120 may dwarf differences in V1V2-specific binding.

We also probed the V1V2-specific reactivity of human plasma samples from HIV-1 positive donors (n = 17) to gp70 V1V2 WT and K-M (Fig. 3B). Consistent with HIVIG reactivity, plasma samples from HIV+ individuals showed higher reactivity to gp70 V1V2 K-M as compared to WT (*P <* 0.001, paired Wilcoxon test), perhaps indicating that V2i-like Abs are more prevalent in plasma than V2p-like Abs as previously suggested [16]. There was no significant difference in plasma Ab binding to gp70 V1V2 WT after heat-denaturation, yet binding to gp70 V1V2 K-M decreased significantly after denaturation (*P =* 0.0004, paired Wilcoxon test), suggesting that a substantial portion of V1V2-specific mAbs recognizing gp70 V1V2 K-M is conformation-dependent in many of the donor samples. Together, these results demonstrate that K-M imparts altered V1V2 antigenicity as evaluated by mAbs and polyclonal Ab samples, in a conformation-dependent manner.

### 2.2 Differential antigenicity results in differential immunogenicity

A key assumption of structure-based immunogen design is that an immunogen that faithfully recapitulates the conformation of relevant epitopes (antigenicity) will induce Ab responses recognizing those epitopes (immunogenicity). To determine whether K-M antigenicity was sufficient to preferentially elicit conformation-dependent V2q- or V2i-like cross-reactive Ab responses, we evaluated the immunogenicity of K-M variants in two mouse models wherein murine MHC class II complexes are replaced with chimeric human/murine MHC class II, comprising the antigen-binding domains from the human leukocyte antigen (HLA) DRB1*04:01 allele [38] (DR4-restricted) or with human DRB1*15:01 [39] (DR2-restricted). While not strictly necessary, the HLA-restricted mouse models enable evaluation of immunogenicity in the context of T cell help based on known human HLA alleles.

#### 2.2.1 Immunization with gp70 V1V2 K-M but not WT elicits a broadly reactive antibody response in a DR4-restricted mouse model

**Fig. 4:**
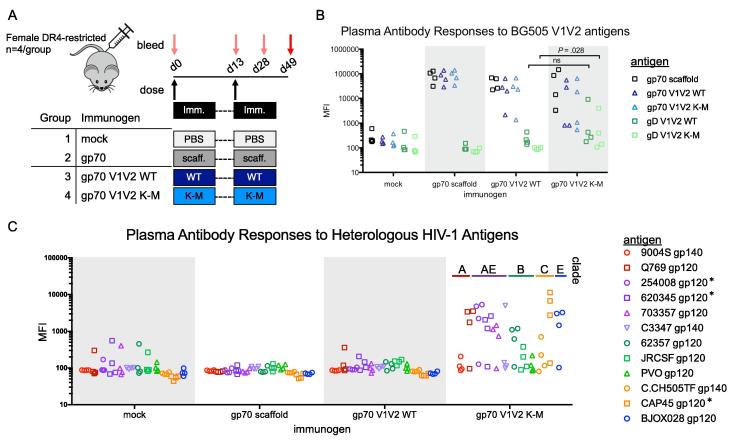
Immunogenicity of gp70 V1V2 variants in a DR4-restricted mouse model. A. Schematic of study design. Groups were immunized twice with PBS (mock), gp70 scaffold, or gp70 V1V2 WT/K-M variants. B-C. Plasma antibody responses to immunogens and autologous gD V1V2 antigens (B) and heterologous gp120/gp140 antigens (C) at day 49. Each point represents the mean of triplicate measurements for each mouse. In panel C, heterologous antigens are colored by HIV-1 clade. Two-tailed Mann-Whitney tests were performed in GraphPad prism, with comparisons made and unadjusted exact *P* value shown in panel B. Asterisks to the right of antigen names in the panel C legend denote a significant difference between gp70 V1V2 WT and K-M immunized groups (unadjusted exact *P* value = 0.0286).

To compare the immunogenicity of gp70 V1V2 WT and K-M in the context of a potent adjuvant, four groups of female DR4-restricted mice (n = 4/group) were dosed twice with PBS (mock), gp70 scaffold, gp70 V1V2 WT and gp70 V1V2 K-M with complete Freund’s adjuvant (dose 1) and incomplete Freund’s adjuvant (dose 2) (Fig. 4A). Unsurprisingly, all experimental groups developed robust responses to the gp70 scaffold (Fig. 4B), which is derived from a murine leukemia virus protein. To parse V1V2-specific from gp70 scaffold-specific responses, we examined plasma Ab responses to gD V1V2 antigens. Immunization with gp70 V1V2 WT (Group 3) and gp70 V1V2 K-M (Group 4) both elicited Abs to gD V1V2 WT, yet only gp70 V1V2 K-M immunized mice developed a response to gD V1V2 K-M (unadjusted *P* = 0.028, Mann-Whitney test). Most strikingly, mice immunized with gp70 V1V2 K-M developed highly cross-reactive Ab responses to the panel of heterologous gp120/gp140 antigens from five HIV-1 clades [40], whereas no cross-reactive antibodies were observed among scaffold or gp70 V1V2 WT immunized groups (Fig. 4C). Thus, gp70 V1V2 K-M displayed a markedly different immunogenicity profile than gp70 V1V2 WT; mice immunized with gp70 V1V2 WT developed narrow responses to an autologous gD V1V2 WT, while gp70 V1V2 K-M immunized mice developed responses to both gD V1V2 WT and K-M, which was associated with cross-reactive responses to heterologous gp120/gp140 antigens.

#### 2.2.2 Defining the effects of scaffold, epitope-restriction and vaccination regimen in a DR2-restricted mouse immunization model

**Fig. 5:**
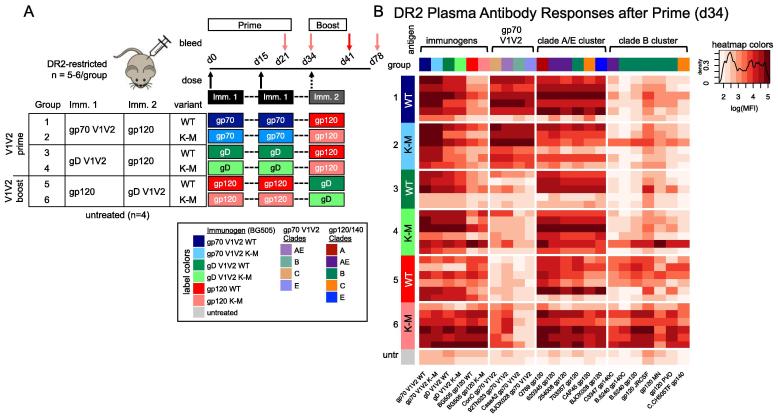
Immunogenicity of gp70 V1V2, gD V1V2 and gp120 WT and K-M variants in a DR2-restricted mouse model. A. Schematic of study design. Groups were immunized twice with a primary immunogen (Imm. 1, prime), followed by a third immunization with a secondary immunogen (Imm. 2, boost). V1V2 prime groups (Imm. 1 = gp70- or gD-V1V2; groups 1–4) were subsequently boosted with gp120, while V1V2 boost groups (Imm. 1 = gp120; groups 5–6) were immunized first with gp120 followed by a gD-V1V2 boost. Groups 1, 3, and 5 received only WT immunogens, and groups 2, 4, and 6 received only K-M immunogens. B. Heatmap of DR2 plasma antibody responses as measured by a customized multiplex bead immunoassay (after prime phase, day 34) to antigens grouped according to labeled brackets (immunogens, heterologous gp70 V1V2, and heterologous gp120/gp140 antigens split into hierarchical clustering-defined clade A/E and clade B clusters). Rows (colored by immunization group and Imm. 1) show responses by mouse, and each column represents responses to a single antigen. Pixels are colored by the log-transformed median fluorescence intensity (MFI) averaged over three replicate measurements.

To explore the generalizability of K-M elicited Ab breadth, we next assessed the effects of additional immunization parameters on the magnitude and breadth of V1V2-specific Ab responses elicited by K-M immunogens. In total, six groups (n = 5–6/group) of DR2-restricted mice were primed with gp70 V1V2 WT/K-M, gD V1V2 WT/K-M or gp120 WT/K-M, with the objectives of comparing the effects of scaffold (gp70 vs. gD) and epitope-focusing (gp70/gD-V1V2 vs. gp120) (Fig. 5A). Groups were subsequently boosted to examine different vaccination regimens with respect to immunization sequence (V1V2 prime or V1V2 boost). Ab responses were monitored at four time points [day 34 (Fig. 5B); days 21, 41 and 78 (Fig. S4)], and compared to serum from naïve mice (n = 4) as negative controls. Given the high variance of Ab responses within immunization groups (e.g. with non- and low-responders), as well as the large number of antigens (24) and immunization groups, this study was not powered to achieve statistical significance with stringent corrections for multiple comparisons. Below, we present observations with unadjusted *P* values to aid with interpretation.

#### 2.2.3 Immunization with gD V1V2 K-M and gp120 K-M variant elicits more robust and cross-reactive Ab responses to a cluster of heterologous gp120/gp140 antigens

**Fig. 6:**
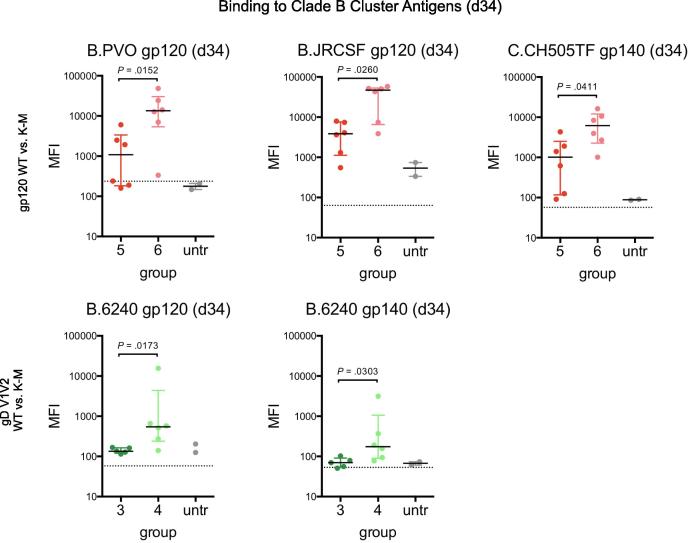
Plasma Ab responses in gp120 and gD V1V2 immunized mice to individual antigens in the Clade B cluster. Responses after prime to a subset of individual gp120/gp140 antigens from the Clade B cluster are shown for mice immunized with gp120 WT or K-M (top; groups 5–6) and gD V1V2 WT or K-M (bottom; groups 3–4). Each point represents the mean of three replicate MFI measurements for each mouse. Bar and whiskers denote median and interquartile range for each group, and dotted lines indicate background fluorescence values for each antigen-coated bead. Two-tailed Mann-Whitney tests were performed in GraphPad prism for the comparisons shown, and unadjusted *P* values are indicated.

Hierarchical clustering was employed in order to holistically analyze plasma Ab responses against this set of 24 immunogens/antigens, and this analysis consistently identified four antigen clusters through day 41: scaffolded V1V2 immunogens, heterologous gp70 V1V2 antigens, and two distinct sets of heterologous gp120/gp140 antigens (clustering dendrograms shown in Fig. S5; antigens grouped by cluster in Figs. 5B and S4). The two distinct sets of heterologous gp120/gp140 antigens exhibited differential reactivity profiles, and were comprised of either primarily clade B antigens (“clade B cluster”) or clade A/AE/E antigens (“clade A/E cluster”). In this model, all groups exhibited strong Ab responses to the clade A/E cluster, while Ab responses to clade B cluster antigens were more variable. Initial immunization with gp120 (groups 5 and 6) resulted in the strongest responses to clade B cluster antigens, and immunization with the K-M variant resulted in the most robust Ab responses (Fig. 6, top). Additionally, immunization with gD V1V2 K-M (group 4) also resulted in stronger responses than did immunization with gD V1V2 WT (group 3) for strain B.6240 antigens (Fig. 6, bottom). Responses to clade B cluster antigens were not appreciably different between gp70 WT and K-M groups.

#### 2.2.4 Immunization with gp120 K-M elicits V1V2-specific Ab breadth and CD4bs-directed responses

**Fig. 7:**
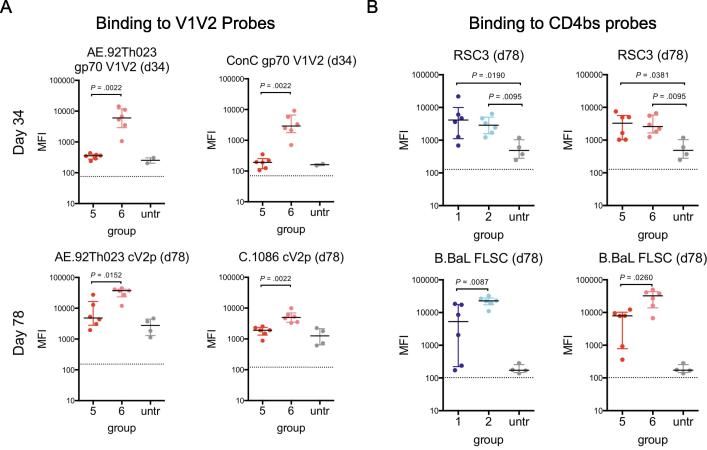
Plasma Ab responses to epitope-specific probes. A. Plasma Ab responses to V1V2 probes at day 34 (top) and day 78 (bottom) for gp120-primed groups (groups 5–6). B. Plasma Ab responses to resurfaced stabilized core (RSC3; binds preferentially to CD4bs bnAbs) and full-length single-chain gp120-CD4 complex (FLSC; exposes CD4-inducible epitope) at day 78 for gp70 V1V2-primed (left; groups 1–2) and gp120-primed (right; groups 5–6) groups. Each point represents the mean of three replicate MFI measurements for each mouse. Bar and whiskers denote median and interquartile range for each group, and dotted lines indicate background fluorescence values for each antigen-coated bead. Two-tailed Mann-Whitney tests were performed in GraphPad prism for the comparisons shown, and unadjusted *P* values are indicated.

We next parsed the V1V2-specificity of gp120 K-M elicited Ab responses by assessing plasma Ab responses to V1V2 probes (Fig. 7A). Priming with gp120 K-M before a gD V1V2 boost was sufficient to elicit V1V2-specific responses as assessed by heterologous gp70 V1V2 antigens while responses after priming with gp120 WT (group 5) was minimal (Fig. 7A, top). V1V2-specific breadth as a result of the gp120 K-M prime persisted, as observed in day 78 plasma Ab reactivity to cyclic V2 peptides (cV2p) (Fig. 7A, bottom). Thus, altered V1V2 K-M antigenicity, even in the context of a full-length gp120 immunogen can translate to altered V1V2-specific immunogenicity.

We also assessed induction of CD4bs- and CD4i- specific Abs. Groups primed with gp70 V1V2 WT/K-M and boosted with gp120 WT/K-M (groups 1 and 2) developed comparable responses to resurfaced stabilized core (RSC3) [41], which preferentially reacts with CD4bs bnAbs (Fig. 7B, top), suggesting the presence of CD4bs-specific Abs. Priming with gp120 K-M (group 6) as well as boost with gp120 K-M in the context of a gp70 V1V2 K-M prime (group 2) resulted in markedly higher plasma Ab reactivity at day 78 to a full-length single-chain clade B.BaL gp120-CD4 antigen (FLSC), which exposes the CD4i epitope [42] (Fig. 7B, bottom), relative to groups with the corresponding WT vaccination regimen. Differential binding to FLSC could be reflective of Ab cross-reactivity to a heterologous clade B antigen, though there may be enhanced reactivity to the CD4i epitope exposed in a more open gp120 conformation.

#### 2.2.5 Immunogen scaffold and regimen modulate the robustness of response

**Fig. 8:**
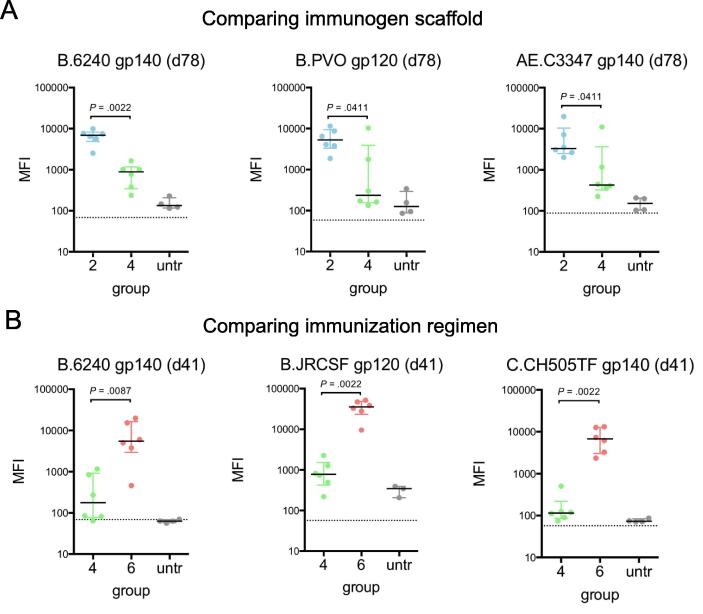
Comparisons of immunogen scaffold and immunization regimens. A. Plasma Ab responses to gp120/gp140 antigens from the clade B cluster for gp70 V1V2 K-M (blue; group 2) and gD V1V2 K-M (green; group 4), to compare the use of gp70 or gD scaffold. B. Plasma Ab responses to gp120/gp140 antigens from the clade B cluster for gD V1V2 K-M-primed (green; group 4) or gp120 K-M-primed (pink; group 6), to compare V1V2/gp120 prime/boost and gp120/V1V2 prime/boost regimens. Each point represents the mean of three replicate MFI measurements for each mouse. Bar and whiskers denote median and interquartile range for each group, and dotted lines indicate background fluorescence values for each antigen-coated bead. Two-tailed Mann-Whitney tests were performed in GraphPad prism for the comparisons shown, and unadjusted *P* values are indicated. (For interpretation of the references to colour in this figure legend, the reader is referred to the web version of this article.)

Groups immunized with a gp70 V1V2 prime exhibited persistent and more robust Ab responses to clade B cluster antigens than groups primed with gD V1V2 for both WT and K-M groups (Fig. S4C). This effect was most apparent comparing Ab responses from K-M immunized V1V2-prime groups (group 2 and group 4) and persisted through the final timepoint at day 78 (Fig. 8A). That differential robustness was observed in both WT and K-M groups suggests that use of the highly immunogenic gp70 scaffold, as observed in the gp70-specific responses in the DR4 model described above (Fig. 4B), may contribute to overall immunogenicity of the vaccine regimen, as compared to use of the gD scaffold.

To directly compare the effect of gp120 prime versus gp120 boost, mice with a gp120 prime followed by gD V1V2 boost (groups 5–6) exhibited higher magnitude responses to the clade B cluster, as compared to mice with the reverse regimen (gD V1V2 prime with gp120 boost; groups 3–4) in both WT and K-M contexts (Fig. S4B-C). Again, this effect was most apparent for K-M variants (Fig. 8B), and overall these observations suggest that there may be a benefit from immunizing first with full-length gp120 rather than with gD V1V2.

## 3 Discussion

In this study, we extended earlier observations that the HIV-1 V1V2 K-M sequence variant exhibits altered antigenicity, and evaluated its antigenicity and immunogenicity in the context of two epitope-restricted immunogens (gp70 V1V2 and gD V1V2) and full-length monomeric gp120. V1V2 K-M variants have increased binding to V2q and V2i mAbs, suggesting an increased propensity for V1V2 K-M to adopt the V2C β-strand conformation recognized by these mAb classes, and reduced binding to V2p mAbs suggesting decreased prevalence of alternative α-helix-coil conformations. The main objective of this study was to determine whether differential V1V2 K-M antigenicity translates to differential immunogenicity. That K-M variants elicited Ab responses with greater magnitude and breadth in two mouse models, and in various immunogen and regimen contexts suggests that V1V2 conformation may influence the cross-reactivity of the Abs generated, as well as the epitope conformation recognized.

We characterized V1V2 K-M immunogenicity profiles in mouse models that allow observation of a link between V1V2 conformation/antigenicity and immunogenicity, despite not being wholly representative of human immunity. Further determination of K-M immunogenicity profiles in the context of adjuvants more likely to be used in humans and in *in vivo* models more relevant to human Ab responses to HIV-1 will be required to further translate our observations. Like other formulations, Freund’s adjuvant, which was selected based on its potency and wide use in mouse immunization studies, may have an effect on immunogen conformation [43], but was not investigated here.

The main readout for immunogenicity here was plasma Ab reactivity to a recombinant antigen panel. Though widely employed to differentiate specificity and breadth of Ab responses [40], antigen binding readouts are not fully informative with respect Ab function. Beyond neutralization, highly cross-reactive V2i-like Ab responses that may be elicited by K-M immunogens have the potential to more broadly exert antiviral activity such as antibody dependent cellular cytotoxicity and phagocytosis, given appropriate Fc characteristics such as IgG subclass and glycosylation. Thus, it will be important to assess how differential immunogenicity may translate to differential Ab functionality, and comprehensive analysis of the functionality of K-M elicited Abs should consider neutralizing and extra-neutralizing functions.

V1V2 immunogenicity must also be considered in the context of glycosylation, as the heavy glycosylation of the V1V2 loops can modulate Ab recognition [45]. However, the immunogens used in this study were likely heterogeneous with respect to glycoform composition, as specialized cell lines or media additives were not used in immunogen preparation. Though we cannot exclude effects of glycan occupancy or glycoform composition, we previously showed that enhanced recognition of K-M variants by V2i mAbs was not glycan-dependent [32], suggesting that differences in structure rather than glycosylation drive differential antigenicity, and this perhaps extends to the immunogenicity of K-M variants.

Given the intrinsic metastability, and thus, conformational plasticity of viral membrane glycoproteins such as the HIV-1 envelope trimer, there remains active debate about which epitopes/conformations to target for vaccine design. Indeed, there may be several solutions. For example, as has been suggested for approaches targeting the HIV-1 fusion peptide [46], it may be more feasible to aim for an Ab response that is permissive to structural variation in the cognate epitope to decrease the entropic cost of Ab:epitope binding. Yet, eliciting an Ab response against flexible targets may be challenging, motivating an approach to present plastic epitopes in stabilized, vulnerable conformations. Undoubtedly, a successful solution will depend on the epitope and the Ab’s mechanism of recognition and function. As K-M variants stabilize the V2C β-strand epitope recognized by cross-reactive V2i mAbs, we hypothesize that K-M may better elicit V2i-like cross-reactive Abs with the potential to broadly recruit immune effector cells rather than V2p-like mAbs which may exhibit narrower reactivity [27], [28]. More generally, our results suggest that conformational nuances, such as fine-tuning the V2 conformational balance between α-helix and β-strand, may modulate the cross-reactivity associated with conformational selectivity of the elicited Ab response.

## 4 Methods

### 4.1 General

DNA for wildtype BG505 gp70 V1V2, gD V1V2, and gp120 constructs were purchased as gBlock fragments from Integrated DNA Technologies (IDT). Oligonucleotides were purchased from IDT. Phusion DNA Polymerase, DNA restriction, ligation enzymes and Gibson Assembly master mix were purchased from New England Biolabs (NEB), and DNA purification/gel-extraction were performed using Geneaid or Macherey-Nagel kits. Site-directed mutagenesis was performed with Turbo pfu polymerase (Agilent). All DNA sequences were verified by Genewiz, Inc. Chemically competent *Escherichia coli* strain DH5α (NEB) were used as hosts for molecular cloning, and DNA plasmid purification from bacteria was performed using QIAPrep Spin Miniprep (Qiagen) or PureYield Plasmid Maxiprep (Promega) kits.

The following reagents were obtained through the NIH AIDS Reagent Program, Division of AIDS, NIAID, NIH: CH58 from Drs Barton F. Haynes and Hua-Xin Liao [27], [47]; 697-30D from Dr. Susan Zolla-Pazner [22]; and anti-HIV Immune Globulin (HIVIG), Catalog #3957. mAbs 830A and 2158 were provided by Dr. Susan Zolla-Pazner, and DNA for CAP228-16H/19F and CAP228-3D Abs were provided by Dr. Lynn Morris. DNA for heavy and light variable regions for PG9 and PG16 were purchased as gBlocks (IDT) and cloned into CMV vectors. Patient-derived HIV-1 positive human plasma samples were obtained from SeraCare.

### 4.2 Preparation of DNA constructs, protein production and purification

Mammalian codon optimized wildtype BG505 gp70 V1V2, gD V1V2 and gp120 were cloned into a CMV vector with C-terminal avi 6xhis tags. Site-directed mutagenesis to introduce the K155M mutation in each was performed following the QuikChange site-directed mutagenesis protocol (Agilent).

Proteins were expressed via transient transfection in HEK293F cells using polyetheleneimine (PEI) (Polysciences 23966) as previously described [32] or in ExpiHEK293F cells using the ExpiFectamine 293 Transfection Kit (ThermoFisher A14525). Supernatant was harvested after 5–7 days of culturing via centrifugation at 9000g for 15 min and filtration using 0.22 μm Steritop or Steriflip filter units (Millipore). Scaffolded V1V2 and gp120 proteins were purified using ion metal affinity chromatography (IMAC) using a nickel-charged IMAC Sepharose 6 Fast Flow or HisTrap Excel column (GE Healthcare 17–0921-08 and 17–3712-06). Alternatively, gp120 proteins were purified using agarose-bound Galanthus Nivalus Lectin (Vector Laboratories AL-1243) using phosphate buffered saline (PBS) and cold 0.5 M methyl α-D-*manno*-pyranoside in PBS as equilibration/wash and elution buffer. Abs were purified using CaptureSelect Protein G resin. Eluates were concentrated using Amicon Ultra 15 mL centrifugal filters (Millipore Sigma) prior to size exclusion chromatography on HiLoad 16/600 Superdex 75 pg column (V1V2 scaffolds), HiLoad 16/600 Superdex 200g, or HiPrep 16/60 Sephacryl S-200 HR (GE Healthcare 28989333, 28989335, 17–1166-01) columns in PBS. All purification steps were performed on an Akta Chromatography System (GE Life Sciences).

### 4.3 Customized microsphere immunoassays

Reactivities of mAbs, polyclonal HIVIG, commercial patient plasma and mouse sera to WT/K-M variants and HIV antigens were assessed via customized multiplexed immunoassay as previously described [48]. Briefly, proteins were conjugated to magnetic carboxylated fluorescent beads (Luminex Corp) using a two-step carbodiimide reaction. For binding assays, 500 beads/well of each type of antigen-coated bead were first incubated in 50 μL assay buffer (PBS + 0.1% BSA + 0.05% Tween-20) with mAbs/HIVIG at their desired concentration, or with plasma/serum samples at a 1:1000–1:3000 dilution (determined by titration) in 384-well polystyrene microplates (Corning). To measure Ab and plasma binding to denatured antigens, beads were boiled at 95 °C for 10 min in a thermocycler prior to primary incubation. Samples were incubated for 1 h at room temperature (mAbs); 3 hrs at room temperature or overnight at 4 °C (plasma and serum); with shaking. Plates were then washed 5 times in assay buffer using a BioTek plate washer, followed by incubation in secondary antibody – anti-human Fc-PE or anti-mouse Fc-PE (Southern Biotech 2048-09, 1030-09) – at a concentration of 0.65 μg/mL in 40 μL assay buffer for 1 h at room temperature. All incubations were performed on a microtiter shaker (VWR). Samples were washed and resuspended in 40 μL xMAP Sheath Fluid prior to acquisition on a Bio-Plex array reader (FlexMAP 3D, Luminex). Median fluorescence intensities (MFI) were reported for each bead type, and secondary-only controls were used to assess background fluorescence levels and calculate background-subtracted signals.

### 4.4 *In vivo* immunogenicity studies

Protocols for animal immunizations were approved by the Institutional Animal Care and Use Committee of Dartmouth College, in accordance with the Association for the Assessment and Accreditation of Laboratory Animal Care Guidelines. All efforts were made to minimize animal suffering.

Endotoxin removal from immunogen preparations were performed as previously described [49]. Briefly, 3 rounds of the following procedure were performed. Protein solutions in PBS were incubated with 1% v/v Triton X-114 with rotation at 4 °C for 30 min, followed by incubation at 37 °C for 10 min. Samples were then centrifuged at maximum speed (17,000g) in a tabletop microfuge for phase separation. The liquid phase was decanted and subjected to further rounds of endotoxin removal, or saved for immunizations. Endotoxin quantitation was performed using Pyrochrome Chromogenic Endotoxin Testing Reagents (Associates of Cape Cod Inc.).

For mouse immunizations, 100 μL at a 0.1 mg/mL protein concentration (10 μg protein) were administered subcutaneously with Freund’s Complete Adjuvant (CFA) on day 0 or Freund’s Incomplete Adjuvant (IFA) thereafter. For DR4-restricted mice (Fig. 4A), 4 groups of 7–8 weeks-old female mice (n = 4) were given PBS (mock), gp70 scaffold, gp70 V1V2 WT or gp70 V1V2 K-M at days 0 and 13. DR4-restricted mice were bled via retro-orbital puncture at days 0, 13, 28 and 49 to monitor Ab responses. For DR2-restricted mice (Fig. 5A), immunizations were performed as described above for 6 groups (n = 6, except n = 5 for group 3) of 7–8 week-old gender-balanced groups, and age-matched naïve mice were used as negative controls. Mice were immunized with a primary immunogen during the prime phase on days 0 and 15, followed by immunization with secondary immunogen (‘boost’) on day 34. Blood was collected via retro-orbital puncture at days 21, 34 and 41, and a cardiac puncture terminal bleed was performed at day 78. Plasma was harvested by centrifugation at maximum speed (17,000g) in a tabletop microfuge at room temperature and stored at −20 °C until use.

### 4.5 Antigens used for characterization of murine plasma Ab responses

Heterologous gp120, gp140 and gp70 V1V2 antigens were generously provided by the Collaboration for AIDS Vaccine Discovery (CAVD) Antigen Reagent Program (ARP) [40]. Cyclic V2 peptides were generously provided by Dr. Susan Zolla-Pazner. The following reagent was obtained through the NIH AIDS Reagent Program, Division of AIDS, NIAID, NIH: RSC3 from Drs. Zhi-Yong Yang, Peter Kwong, Gary Nabel (cat #12042) [41], [50]. Full-length gp120-CD4 (FLSC) was generously provided by Drs. Anthony DeVico, Timothy Fouts, and George Lewis.

### 4.6 Statistical methods

Regressions and significance testing were performed using the Prism GraphPad software v. 6.0 h. Binding curves were subjected to nonlinear sigmoidal regression, where bottom values were constrained to the background value of the assay performed, and coefficients were weighted by 1/Y^2^. Paired nonparametric Wilcoxon matched-pairs signed rank test was performed to analyze effects of heat denaturation on antigen binding by human plasma samples. Two-tailed Mann-Whitney tests were performed on all other comparisons.

Heatmap analysis of plasma Ab responses was performed in the R statistical software environment. Ab binding data for each time point (log-transformed MFI for each mouse to each antigen averaged over 3 technical replicates) were subjected to unsupervised hierarchical clustering using the complete linkage method.

## Declaration of Competing Interest

The authors declare that they have no known competing financial interests or personal relationships that could have appeared to influence the work reported in this paper.
