# Supplementary material for: Induction of cross-reactive HIV-1 specific antibody responses by engineered V1V2 immunogens with reduced conformational plasticity
Source: Vaccine. 2020 Apr 16;38(18):3436–46. doi: 10.1016/j.vaccine.2020.03.010 (PMC7132531; doi:10.1016/j.vaccine.2020.03.010)
Supplement: Supplementary data 1 [file mmc1.docx]

**Supplementary Information**

**Induction of cross-reactive HIV-1 specific antibody responses by engineered V1V2 immunogens with reduced conformational plasticity**

Jennifer I. Lai_a_, Susan K. Eszterhas_a_, Seth A. Brooks_a_, Chengzi Guo_a_, Susan Zolla-Pazner_b_, Michael S. Seaman_c_, Chris Bailey-Kellogg_d_, Karl E. Griswold_a_, Margaret E. Ackerman_a,e_*

| **Supplemental Figures/Tables** |  |
| --- | --- |
| Supplemental Figure 1 | K155M mapped onto the structure of BG505 SOSIP |
| Supplemental Figure 2 | Binding of V1V2 K-M variants to additional V1V2 mAbs |
| Supplemental Figure 3 | Binding orientation of CD4bs mAbs relative to V1V2 loops mapped on BG505 SOSIP |
| Supplemental Figure 4 | Heatmaps of DR2 plasma antibody responses |
| Supplemental Figure 5 | Hierarchical clustering of DR2 plasma antibody responses |
| Supplemental Figure 6 | Plasma antibody responses to heterologous clade B cluster antigens after gp70 V1V2 prime and gp120 boost |
| Supplemental Table 1 | Sequences of BG505 V1V2 scaffold and gp120 variants |
| Supplemental Table 2 | EC50 values for binding of WT and K-M variants to V1V2 mAbs with 95% confidence intervals |
| Supplemental Table 3 | EC50 values for gp120 WT/K-M binding to CD4bs and CD4i mAbs with 95% confidence intervals |

**Supplemental Figure 1: K155M mapped onto the structure of BG505 SOSIP.** Each monomer of gp120 is shown in cartoon (gray), surface rendering (gray), or cartoon and mesh (wheat with V1V2 loops in orange). K155M is shown in red with surface rendering, and while minimally visible in the top view (left), can be seen below the V1V2 apex in the side view (right). (PDBID: 5V8M)

**Supplemental Figure 2: Binding of V1V2 K-M variants to additional V1V2 mAbs.** Binding of K-M variants was measured to V2q [PG16 (light blue)] and V2p [16H, 19F, 3D (red)] mAbs. Dotted lines on y-axes represent background signal from no antibody controls, and error bars represent standard deviation of triplicate measurements. Nonlinear fit lines calculated in GraphPad Prism are shown for data where calculated EC50 values are non-ambiguous.

**Supplemental Figure 3: Binding orientation of CD4bs mAbs relative to V1V2 loops mapped on BG505 SOSIP.** Crystal structures [PDB IDs: 3NGB (VRC01), 2NY7 (b12), 5V8M (3BNC117/SOSIP), 3hI1 (F105)] were aligned such that gp120s were aligned with the BG505 SOSIP structure from 5v8m. Antibodies are shown in cyan (b12 Fab), green (VRC01 Fab), blue (3BNC117 scFv) and yellow (F105 Fab), while one gp120 protomer is colored in wheat, and V1V2 loops colored in orange.

**Supplemental Figure 4: Heatmaps of DR2 plasma antibody responses.** Plasma antibody responses were measured against antigen groups clustered according to labeled brackets (immunogens, gp70 V1V2, clade A/E cluster, and clade B cluster) at day 21 (A), day 34 (B) and day 78 (C). Each row (colored by immunization group and primary immunogen) shows responses by mouse, and each column represents responses to a single antigen. Each pixel shows the log-transformed median fluorescence intensity (MFI) averaged over three replicate measurements.

**Supplemental Figure 5: Hierarchical clustering of DR2 plasma antibody responses.** Plasma antibody responses at day 21 (left) and day 41 (right) were hierarchically clustered by antigen (columns), consistently revealing four major antigen groups demarcated by solid white lines: heterologous gp70 V1V2, heterologous clade A/E cluster, heterologous clade B cluster, and autologous immunogens (imm). Ab responses to autologous BG505 gp120 immunogens are marked by dotted white lines, as these responses clustered with the antigens from the heterologous clade B cluster.

**Supplemental Figure 6: Plasma antibody responses to heterologous clade B cluster antigens after gp70 V1V2 prime and gp120 boost.** Group 1-2 plasma antibody responses at day 78 after gp120 boost to a subset of gp140 antigens from the heterologous clade B cluster. Each point represents the mean of three replicate MFI measurements for each mouse. Bar and whiskers denote median and interquartile range for each group, and dotted lines indicate background fluorescence values for each antigen-coated bead.

**Supplemental Table 1: Sequences of BG505 V1V2 scaffold and gp120 variants^+^**

|  | Amino acid sequence |
| --- | --- |
| gp70 V1V2 WT | AAPGSSPHQVYNITWEVTNGDRETVWAISGNHPLWTWWPVLTPDLCMLALSGPPHWGLEYQAPYSSPPGPPCCSGSSGSSAGCSRDCDEPLTSLTPRCNTAWNRLKLDQVTHKSSEGFYVCPGSHRPREAKSCGGPDSFYCASWGCETTGRVYWKPSSSWDYITVDNNLTTSQAVQVCKDNKWCNPLAIQFTNAGKQVTSWTTGHYWGLRLYVSGRDPGLTFGIRLRYQNLGPRVPIGPNPVLADQLSLPRPNPLPKPAKSPPASLKPCVKLTPLCVTLQCTNVTNNITDDMRGEL**K**NCSFNMTTELRDKKQKVYSLFYRLDVVQINENQGNRSNNSNKEYRLINCNTSAITQACPKVS*GGGGSGGGGHVLNDIFEAQKIEWHETGHHHHHH* |
| gp70 V1V2 K-M | AAPGSSPHQVYNITWEVTNGDRETVWAISGNHPLWTWWPVLTPDLCMLALSGPPHWGLEYQAPYSSPPGPPCCSGSSGSSAGCSRDCDEPLTSLTPRCNTAWNRLKLDQVTHKSSEGFYVCPGSHRPREAKSCGGPDSFYCASWGCETTGRVYWKPSSSWDYITVDNNLTTSQAVQVCKDNKWCNPLAIQFTNAGKQVTSWTTGHYWGLRLYVSGRDPGLTFGIRLRYQNLGPRVPIGPNPVLADQLSLPRPNPLPKPAKSPPASLKPCVKLTPLCVTLQCTNVTNNITDDMRGEL**M**NCSFNMTTELRDKKQKVYSLFYRLDVVQINENQGNRSNNSNKEYRLINCNTSAITQACPKVS*GGGGSGGGGHVLNDIFEAQKIEWHETGHHHHHH* |
| gD V1V2 WT | KYALADASLKMADPNRFRGKDLPVLDQLLEVPCVKLTPLCVTLQCTNVTNNITDDMRGEL**K**NCSFNMTTELRDKKQKVYSLFYRLDVVQINENQGNRSNNSNKEYRLINCNTSAITQACPKVS*GGGGSGGGGHVLNDIFEAQKIEWHETGHHHHHH* |
| gD V1V2 K-M | KYALADASLKMADPNRFRGKDLPVLDQLLEVPCVKLTPLCVTLQCTNVTNNITDDMRGEL**M**NCSFNMTTELRDKKQKVYSLFYRLDVVQINENQGNRSNNSNKEYRLINCNTSAITQACPKVS*GGGGSGGGGHVLNDIFEAQKIEWHETGHHHHHH* |
| gp120 WT | AENLWVTVYYGVPVWKDAETTLFCASDAKAYETEKHNVWATHACVPTDPNPQEIHLENVTEEFNMWKNNMVEQMHTDIISLWDQSLKPCVKLTPLCVTLQCTNVTNNITDDMRGEL**K**NCSFNMTTELRDKKQKVYSLFYRLDVVQINENQGNRSNNSNKEYRLINCNTSAITQACPKVSFEPIPIHYCAPAGFAILKCKDKKFNGTGPCPSVSTVQCTHGIKPVVSTQLLLNGSLAEEEVMIRSENITNNAKNILVQFNTPVQINCTRPNNNTRKSIRIGPGQAFYATGDIIGDIRQAHCTVSKATWNETLGKVVKQLRKHFGNNTIIRFANSSGGDLEVTTHSFNCGGEFFYCNTSGLFNSTWISNTSVQGSNSTGSNDSITLPCRIKQIINMWQRIGQAMYAPPIQGVIRCVSNITGLILTRDGGSTNSTTETFRPGGGDMRDNWRSELYKYKVVKIEPLGVAPTRAKRRVVGTGLK*GGGGSGGGGHVLNDIFEAQKIEWHEHHHHHH* |
| gp120 K-M | AENLWVTVYYGVPVWKDAETTLFCASDAKAYETEKHNVWATHACVPTDPNPQEIHLENVTEEFNMWKNNMVEQMHTDIISLWDQSLKPCVKLTPLCVTLQCTNVTNNITDDMRGEL**M**NCSFNMTTELRDKKQKVYSLFYRLDVVQINENQGNRSNNSNKEYRLINCNTSAITQACPKVSFEPIPIHYCAPAGFAILKCKDKKFNGTGPCPSVSTVQCTHGIKPVVSTQLLLNGSLAEEEVMIRSENITNNAKNILVQFNTPVQINCTRPNNNTRKSIRIGPGQAFYATGDIIGDIRQAHCTVSKATWNETLGKVVKQLRKHFGNNTIIRFANSSGGDLEVTTHSFNCGGEFFYCNTSGLFNSTWISNTSVQGSNSTGSNDSITLPCRIKQIINMWQRIGQAMYAPPIQGVIRCVSNITGLILTRDGGSTNSTTETFRPGGGDMRDNWRSELYKYKVVKIEPLGVAPTRAKRRVVGTGLK*GGGGSGGGGHVLNDIFEAQKIEWHEHHHHHH* |

^+^V1V2 loops are underlined, K-M positions are bolded, and linkers/tags are italicized.

**Supplemental Table 2: EC50 values for binding of WT and K-M variants to V1V2 mAbs with 95% confidence intervals**

**Supplemental Table 3: EC50 values for gp120 WT/K-M binding to CD4bs and CD4i mAbs with 95% confidence intervals**

***** mAb breadth categories from Georgiev et al. *Curr Opin HIV AIDS* (2013)
